# Editorial: Comparative Studies of Energy Homeostasis in Vertebrates

**DOI:** 10.3389/fendo.2018.00291

**Published:** 2018-06-05

**Authors:** Maximilian Michel

**Affiliations:** Institute of Zoology, University of Cologne, Cologne, Germany

**Keywords:** energy homeostasis, melanocortins, leptin, food intake regulation, energy expenditure

**Editorial on the Research Topic**

**Comparative Studies of Energy Homeostasis in Vertebrates**

Energy homeostasis of an organism is the sum of processes integrating energy intake with resource allocation. Its central control mechanisms are essential to an animal’s life history and govern daily activity, such as searching for food, reproduction, etc. The first major inroads into our understanding of the genetic basis of energy homeostasis were made by the fortuitous finding of five monogenic obesity strains in mouse breeding experiments (1) *obese*, (2) *diabetes*, (3) *agouti*, (4) *fat*, and (5) *tubby* (1). The genes underlying these strains were eventually cloned, laying the groundwork for the analysis of the neural circuitry which underlies energy homeostasis in mice [for recent reviews, please see Ref. (2–4)]. This research quickly led to the discovery of the core genes involved in energy homeostasis in obese human patients such as the leptin system (5, 6) which is mutated in the *obese* and *diabetes* mouse strains or the melanocortin 4 receptor system (7, 8) which is impacted in the *agouti* mouse strain. By and by, the key genes involved in mammalian energy homeostasis were cloned and found to be conserved not only across mammals but also across all vertebrates. However, while the genes are conserved, the anatomical and functional data on these genes varies between species from comparable to variable. In contrast, research into the biochemical basis of energy acquisition and utilization showed clearly that large swaths of the underlying building blocks are highly conserved (9). It follows that the basic building blocks which play a role in energy homeostasis are largely conserved, for example, lipid handling by adipose tissue or glucose handling by the liver. In contrast, the central regulation of these processes differs between species. An argument can be made that these differences in regulation are likely dependent on the ecological niche of an individual species [see for example, Ronnestad et al. in this issue]. Understanding what exactly constitutes a conserved building block in energy homeostasis and how these are centrally regulated to allow an animal to exist in a given ecological niche will allow us to (1) gain insight into the evolution of biological diversity; (2) modulate growth behavior in farm animals (domestication as an ecological niche); and (3) ameliorate abnormal homeostasis patterns in humans (from metabolic syndrome on one extreme to anorexia and cancer cachexia on the other). This research topic comprises reviews and a research article focused on recent insight. Of note—this topic is complemented by a concurrent research topic on the “Neuroendocrine Control of Feeding Behavior” (10), which has a strong emphasis on mammalian systems.

The first set of contributions covers lipid and glucose handling in zebrafish. The first review by Steven Farber’s group (Quinlivan and Farber) introduces the mechanisms governing embryonic and larval lipid dynamics in zebrafish around the time of the transition from egg yolk feeding to prey feeding. The authors highlight that the gut is colonized by microbiota following the animals first feeding. They show that during this time, gut microbiota has an immense impact on host absorptive processes. Finally, the authors point out the benefits of the zebrafish model system to carefully study the dynamics of lipid absorption and processing (Quinlivan and Farber). Amnon Schlegel (Schlegel) focuses the discussion on lipoprotein biology. He argues for the use of zebrafish as a model system, since cholesteryl ester transfer proteins, which predispose people as well as zebrafish to atherogenic lipoprotein profiles, are not conserved in rodents. This allows for the study of plaque formation in order to identify dyslipidemia and atherosclerosis modifiers—both chemical and genetic (Schlegel). Staying on the topic of adipose biology, James Minchin’s group (Wafer et al.) reviews adipogenesis, in particular its master regulator peroxisome proliferator-activated receptor gamma (PPARG) in fish compared to mammals. Not only is this gene highly conserved, but surprisingly, the authors find that only a single ortholog is present across fish species which suggests a critical role in adipogenesis. Astonishingly, while target genes of PPARG seem to be conserved, ligands are more diverse. This suggests that specifically control of adipose tissue evolved but not the role it plays within the organism (Wafer et al.). Veering away from the control of lipid levels, the next contribution focuses on the control of systemic glucose levels by the pancreatic endocrine cells (Maddison and Chen). The authors showcase the utility of the zebrafish as a model system for beta cell proliferation, differentiation, and regeneration (Maddison and Chen). Taken together, these four contributions argue not only for the utility of the zebrafish as a model system but also that the basic building blocks underlying energy homeostasis are fundamentally conserved across vertebrates.

Conde-Sieira and Soengas expand this in their review on peripheral nutrient sensing systems. Importantly, the authors show that the basic glucosensory and fatty acid sensory mechanisms are conserved from different fish species to mammals. However, they see species-specific differences by how central regulation of energy homeostasis is achieved (Conde-Sieira and Soengas). The topic of central mechanisms is further developed in a paper in the concurrent research topic written by Delgado et al., where the authors specifically look at the hypothalamic integration of signals from glucose and fatty acid sensing systems as well as integration of circadian information and the stress axis to achieve food intake regulation (Delgado et al.). Boswell and Dunn reviewed the central melanocortin system in birds and present data for the conservation of the key aspects of central melanocortin action. Interestingly, the authors point to a little understood connection between the melanocortin system, growth, and food intake in birds (Boswell and Dunn), This effect, also seen in platyfish (11), mice and humans and may be mediated *via* the somatostatin axis (12). Hélène Volkoff in her contribution summarizes what we know to date on the impact of different endocrine systems in fish. While most mammalian peptides and hormones either have an effect on fish metabolic state (when injected) and/or are regulated by metabolic state, the phenotype strongly varies between species. This again suggests that the building blocks supporting metabolic state are intact and conserved but the control mechanisms vary between species. The author points out that this is possibly not all too surprising given the widespread adaptations necessary for more than 30,000 species of fish to find ecological niches (Volkoff). This aspect is further developed by Ronnestad et al., where the authors bring up examples of central control differences in fish with vastly different life history and ecological niches. Specifically, the authors review metabolic control in long-term fasting species (the arctic charr, mouthbrooders, and fasting in aquaculture), throughout life transitions (first feeding to juvenile), in voracious feeders, and in cave-living fish (Ronnestad et al.). Ben Renquist’s group introduces metabolic rate in fish, an essential underlying principle in energy homeostasis. The authors concentrate on variations in proton leak, calcium cycling, and membrane potential and relate this to differences in habitat and ecological niche of the investigated species. They point out that small changes in genes involved in ion gradients and their regulation can correlate with critical biological differences and advantages to the species—allowing the organism to adapt to particularly extreme environmental conditions by changes in metabolic rate (Geisler et al.). The subsequent contribution by Marnix Gorissen (van de Pol et al.) further develops the concept of life history, focusing on what is required for an organism to live and survive in an aquatic niche. With a keen eye on the developments in the mammalian literature, the authors look at insulin and leptin biology in fish, from appetite regulation *via* sexual maturation, salt water adaptation, and glucose/adipose homeostasis to stress (van de Pol et al.).

The next set of reviews focus on mechanisms specifically controlled by leptin signaling. Russell Borski’s group (Deck et al.) starts with an overview of leptin structure and points out that leptin has an anorexigenic effect throughout vertebrates. However, this overall effect is mediated by recruiting different building blocks in different species—mobilization of lipid or glucose stores. The authors then look at the mobilization of energy after osmotic stress, hypoxia, disease, and fasting in fish (Deck et al.). Richard Londraville’s contribution (Londraville et al.) introduces the recent discovery of leptin in birds, where evidence suggests that leptin may not have an endocrine but rather may have an autocrine/paracrine function. The authors embark on an up to-date analysis of leptin receptor and endospanin (an overlapping transcript with modulatory function) evolution and structure, probing databanks based on recent structural insight. This insight can be used to inform future research into leptin function (Londraville et al.). The last contribution in this leptin-specific set of reviews is an original research article from the Denver lab which provides evidence on a scarcely studied aspect of leptin biology: leptin’s role during development (Bender et al.). In *xenopus*, the authors show that leptin levels (secreted by adipose tissue) rise just prior to metamorphosis and stay elevated throughout metamorphosis. They find evidence that leptin mediates cell proliferation and conduct a microarray study which provides a basis for further investigation of leptin’s role and regulation of neurogenesis (Bender et al.). These four articles provide a good overview on what is known about non-mammalian leptin biology and highlight the complex nature of this hormone more than 50 years after the identification of the first leptin phenotypes (13).

The last contribution forms a bridge between nonmammalian energy homeostasis research with the mammalian realm. Here, Stewart Nicol discusses energy homeostasis in monotremes (Nicol). Similar to evidence from birds and fish, leptin does not appear to play a role in adipostasis in monotremes. The author further looks at special life history adaptations, such as seasonality, hibernation, and torpor (Nicol). This is of particular note in relation to the contribution of Ronnestad et al. who discuss the role of seasonality in the arctic charr. An organism’s life history needs to be taken into account when translating insight gained from one organism (for example, a fish or a mouse) to another (such as humans). It is imperative to keep Krogh’s principle of comparative biology in mind (for each problem there will be an animal where it can be most conveniently studied) and the harvest this has brought to our understanding of biology. While historical examples range from action potentials in squid *via* learning in mollusks or the shark rectal gland; relevant examples in the context here could be the study of: (1) metabolic control in voracious feeders and seasonal animals; (2) the thermogenic role of muscle in birds lacking brown adipose; (3) the role of the melanocortin system in body growth in platyfish size morphs and birds; (4) metabolic control of migrating animals (eels, salmon, birds). These contributions clearly show the variety of mechanisms which a single regulatory hormone can control. However, these reviews also point out that (a) a hormone can have one general role (such as the anorexigenic effect of leptin) throughout all vertebrates, while recruiting different building blocks to achieve this effect and (b) that different building blocks (such as control of adipose through PPARG) can be centrally controlled by different ligands depending on the species and likely its ecological niche.

## Author Contributions

The author listed has made a substantial, direct, and intellectual contribution to the work and approved it for publication.

## Conflict of Interest Statement

The author declares that the research was conducted in the absence of any commercial or financial relationships that could be construed as a potential conflict of interest.
